# Road of no return — loss of TP53 paves a defined evolution path from gastric preneoplasia-to-cancer

**DOI:** 10.20892/j.issn.2095-3941.2023.0435

**Published:** 2024-02-05

**Authors:** Liwei An, Yi Han, Shi Jiao, Zhaocai Zhou

**Affiliations:** 1 State Key Laboratory of Genetic Engineering, Zhongshan Hospital, School of Life Sciences, Fudan University, Shanghai 200438, China; 2 Department of Stomatology, Shanghai Tenth People’s Hospital, Department of Biochemistry and Molecular Biology, Tongji University School of Medicine, Shanghai 200072, China

Gastric cancer (GC), the fifth- and fourth-leading causes of morbidity and mortality worldwide, still lacks effective diagnoses and treatment. Moreover, China accounts for nearly 50% of the newly diagnosed and death cases of GC globally^1^. Being directly exposed to external food intake, extremely acidic content, and microorganisms, such as *Helicobacter pylori* and Epstein-Barr virus (EBV), stomach homeostasis is easily perturbed and disrupted. Importantly, the gastric epithelium is composed of multiple cell types, including chief, parietal, pit, and stem cells. The lineages of these gastric epithelial cells are rather plastic, giving rise to a high degree of GC heterogeneity. To date, the complex pathology of GC is poorly understood, especially with respect to the cellular origin, initiation, and molecular evolution of GC, which thwarts efforts to develop strategies for preventing, monitoring, and treating GC^2^.

## TP53-associated GC molecular subtyping

The Cancer Genome Atlas first defined molecular subtypes of GC in 2014, including chromosomal instability [CIN (50%)], microsatellite unstable [MSI (22%)], gnome stable [GS (20%)], and EBV positivity [EBV^+^ (9%)]^3^. The Asian Cancer Research Group performed an analysis of molecular alterations, disease progression, and prognosis in patients with GC, then divided GC into MSI, microsatellite stable with epithelial-mesenchymal transition (MSS/EMT), MSS with TP53-active positivity (MSS/TP53^+^), and MSS with loss of TP53 (MSS/TP53^−^)^4^. The MSS/EMT subtype has the worst prognosis and tends to occur at an earlier stage, whereas the MSS/TP53^+^ active subtype is positively correlated with EBV infection and has a better prognosis and immunotherapy response rate^4^. Despite the well-deciphered genomic landscape of each GC subtype, the exact cellular origin and evolution path remain a long-standing question. Specifically, major driver mutations and the relative contributions of each GC subtype are largely unknown.

Notably, most CIN GC subtypes (∼70%) harbor *TP53* loss of heterozygosity (LOH) mutations, which are positively associated with aggressive phenotypes and are refractory to therapy^3,4^. *TP53* LOH provides a unique viewpoint to explore the dynamic evolution of chromosomal instability induction and further drives preneoplasia tissues into malignant tumors. In support of this notion, a recent study reconstructed the evolutionary trajectories of 38 types of tumors based on an analysis of the mutational processes among 2,658 patients. *TP53* ranks first among genes mutated in early clonal stages with a 12-fold higher frequency across various tumor types^5,6^, indicating that loss of TP53 in an essential step in initiating the tumor evolution process. How a TP53 mutation drives malignant transformation of tumor-initiating cells and stepwise progression to cancer has not been established.

## TP53 loss-driven dynamic tumor evolution *in vivo*

Tumor is thought to arise from the proliferation of a single cell to form a clonal population. Different studies of intra-tumor heterogeneity have suggested different models of tumor evolution. Linear evolution consists of stepwise increases in fitness resulting in periodic ‘‘clonal sweeps’’ that replace the dominant make-up of the tumor. These tumors typically exhibit low degrees of heterogeneity across regions with many public mutations^7^. The neutral or ‘‘big bang’’ model of tumor evolution consists of numerous intermixed subclones that are equally fit due to early bursts of mutational events that persist throughout the life of the lesion^8^. In another model branching evolution is characterized by co-existing subclones that exhibit hierarchical relationships between the clones^9^. Independent of how intra-tumor heterogeneity arises, intra-tumor heterogeneity is inextricably linked to tumor growth differences, aggressiveness, and susceptibility to therapy, and thus an in-depth understanding of this phenomenon would be very valuable^10^.

**Figure 1:**
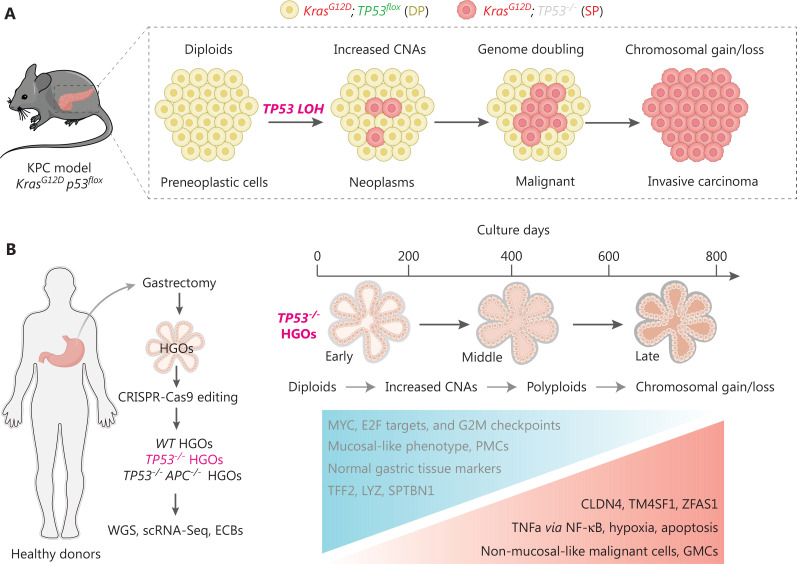
*TP53* inactivation initiates ordered genomic evolution. (A) The Lowe et al. group applied the KPC^LOH^ reporter mouse model harboring both mutated *Kras* (mKate fluorescence) and intact *Tp53* [GFP fluorescence, defined as DP cells (yellow)] and sporadic p53 inactivation resulted in the disappearance of GFP signal [defined as SP cells (red)]. This model allows for simultaneously monitoring TP53 status, genome evolution, and cellular phenotypes *in vivo*. Cartoon presentation is partially adopted^15^. (B) The Curtis’s group utilized human gastric organoids (HGOs) combined with CRISPR-Cas9 technology to generate *TP53^−/−^* HGOs (left panel) and temporally deciphered the genetic alterations with resultant transcriptional reprograms and phenotypes (right panel). Both studies revealed similar orders of genome evolutionary trajectories. *TP53* loss progressively increases CNAs and structural variants, which followed by appearance of aneuploidy and loss/gain of genomic regions.

Tumor initiation and progression are somatic evolutionary processes driven by the accumulation of genetic alterations^11^, some of which confer selective fitness advantages to the host cell^12^. To understand the evolutionary dynamics of cells undergoing stepwise progression to malignancy at the point of *TP53* inactivation, Lowe et al. from Memorial Sloan Kettering Cancer Center and Curtis et al. from Stanford University independently profiled the timing lineage events upon TP53 loss^13,14^. Lowe et al. and Curtis et al. uncovered a deterministic and ordered genetic evolutionary trajectory during preneoplasia. Lowe et al. developed a genetic pancreatic cancer pancreatic ductal adenocarcinoma (PDAC) mouse model with a unique reporter system allowing for tracing genome evolution after sporadic *TP53* inactivation (the KPC^LOH^ model). In the KPC^LOH^ model, the preneoplasia tissue harboring both mutated *Kras* (mKate fluorescence) and intact *TP53* (GFP fluorescence) is designated double-positive (DP), whereas cells involved in induction of TP53 loss in which thge GFP signal vanishes are designated single-positive for mKate fluorescence (SP). Accordingly, the DP-to-SP transition allows the discrimination of cells with biallelic *TP53* inactivation and further lineage-tracing the genomic, transcriptional, and phenotypic analysis of cells after *TP53* LOH by collecting tissues at different time points *in vivo* (**Figure 1A**).

By performing sparse whole-genome and single-cell RNA sequencing of DP and SP cells isolated at distinct stages of transformation, Lowe et al. documented ordered phages of genomic evolution which connect *TP53* LOH to the acquisition of cancer-initiating potential and eventually to PDAC malignancy. Briefly, *TP53* inactivation first induces increased copy-number alterations (CNAs) in diploid cells in an early phase, which is followed by occurrence of rearranged diploid and polyploidy (genome doubling) at a relatively late phase. The emergence of gains and deletions in specified genomic regions represents the last step of genomic evolution (**Figure 1A**). During this process deterministic events include chromatin remodeling (*Mll2* and *Setd2*), axon guidance (*Sema3a* and *Sema3b*), PDAC proliferation or progression (*Il6*, *Shh* and *Cdk8*), and TGF-β signaling (*TgfbrII* and *Bmp5*), which orchestrates the benign-to-malignant transition phenotype^14,15^. Notably, SP cells isolated in early stages display poor colony-forming and tumor-initiating abilities, implying that merely *TP53* LOH coupled with *Kras* activation is not sufficient alone to confer malignant fitness, and the oncogenic properties upon loss of TP53 are acquired over time during genomic evolution^14,15^. In fact, dysregulation of oncogenic signaling, such as *Yap* and *Myc*, are frequently observed long before *TP53* LOH^16^.

## TP53 loss-driven dynamic clonal evolution *in vitro*

Alternatively, Curtis et al. established an *in vitro* system utilizing human gastric organoids (HGOs) combined with CRISPR-Cas9 technology to decipher the causal relationship between genetic alterations with genotypes and phenotypes^13^. Because *TP53* LOH is prevalent in 70% of CIN GC and represents an early event for tumorigenesis^5,6^, Curtis et al. chose to decipher the genetic evolution process driven by TP53 deficiency over a nearly 3-year span of time (**Figure 1B**). Importantly, this study also revealed defined orders of genome evolution featuring progressively increased CNAs, appearance of aneuploidy, and loss/gain of genomic regions along with the time intervals following TP53 deficiency (**Figure 1B**). Specifically, Curtis et al. applied the fraction of genome-altered (FGA) factors, as a marker of aneuploidy, to decipher the dynamic alteration during genome evolution upon loss of TP53. First, Curtis et al. observed that the average FGA score in *TP53^−/−^* HGOs (10%–15%) was much lower than the median FGA detected in CIN GC (34.5% based on cBioPortal), suggesting that additional paralog genome caretakers are required for accelerating the genome evolution *in vivo*. Nevertheless, Curtis et al. compared the FGA between sole TP53 deficiency (*TP53^−/−^* HGOs) and combined *TP53* and *APC* double-knock out (*TP53^−/−^APC^−/−^* HGOs), but observed comparable and plateaued FGA at the end points (approximately 600 days), suggesting that *APC* loss does not fuel this process. Second, Curtis et al. showed that the HGO offspring from the same donor exhibited a distinctive FGA, implying that genome evolution is not strictly determined by genetic background. Of note, the FGAs were even decreased in 2 replicate HGOs from 2 donor patients (D2C1 and D2C3), whereas D2C1 displayed progressive elevation before 400 days of culture, implying that the extremely early evolutionary path may be revisable to some extent or that these early clones are removed due to clonal competition. Third, in addition to the increased FGA frequency along with culture time, the appearance of defined gene locus alterations also follows preferred timing orders. For example, loss of the regions spanning *CDKN2A* (chr9p) and *FHIT/FRA3B* (chr3p), two well-known tumor suppressor genes displaying roughly 41% and 12% mutations in CIN GCs^3^, respectively, were repeatedly detected in the early phase in *TP53^−/−^* HGOs across donors (before 200 days). This finding is consistent with the notion that loss of additional genome caretakers is required for driving the evolutionary path initiated by sole TP53 deficiency.

Next, Curtis et al. also explored the clonal selection, interference, and extinction events initiated by TP53 deficiency (**Figure 1B**). Despite the hotspot mutation frequently observed at the end point of tumorigenesis, the mutational signatures can be quite distinctive in the early phase of evolution. For example, the single-base substitution, 17a/b of *FHIT*, are frequently observed in tumor samples, whereas the initial *TP53^−/−^* HGOs otherwise display substitutions at 1, 5, and 40 positions. Such a discrepancy may be the consequence of convergent evolution and clonal interference further traced the genesis of *FHIT* rearrangements and Curtis et al. showed that some early rearrangements were lost whereas additional alterations occurred in the late stage within this same gene locus. Nevertheless, TP53 deficiency may initiate distinctive sub-clonal evolutionary processes with uncertainty of clonal interference; the clones with endpoint fitness display similar genotypic characters. For example, a stringent selection trajectory includes an early rise of chr4*^−^*, 9q^+^ subclones, followed by replacement with chr19p*^−^*-derived chr8p*^−^*, 9q.2^+^ and 16p*^−^*-altered subclones, which eventually outcompete the chr18q loss that acquired gain of chr20q subclones. Such a clonal evolution trajectory was frequently observed in multiple cultures within the same donor as well as between different donors, again suggesting a preferred order of clonal evolution.

After confirming the genotypic evolution trajectory, Curtis et al. further applied single cell sequencing (sc-RNA) to decipher the reprogramming of transcriptional signatures initiated by TP53 deficiency. As expected, when compared with wild-type HGOs and a normal mucosal gene expression signature, such as *MUC5AC*, *TFF1*, and *TFF2*, those *TP53^−/−^* HGOs stepwise reduce expression of these markers but gradually acquire GC-associated signatures, including increased expression of claudins (*CLDN3/4/7*) and carcinoembryonic antigen (*CEACAM5*/*6*), which phenocopy the molecular events as detected in gastric tumorigenesis models *in vivo*^17,18^. Moreover, the transcriptional comparison between early, middle, and late phases of the same *TP53^−/−^* HGOs consistently identified 13 consistently upregulated genes, such as *CLDN4*, *TM4SF1*, and *ZFAS1*, suggesting these factors are required for the establishment of neoplasia status (**Figure 1B**). GSEA analysis of transcriptional molecular features between highly fit subclones and other clones revealed that signaling pathways, including TNF signaling *via* NF-κB, hypoxia, and apoptosis, were dramatically enforced in these winning subclones (**Figure 1B**), suggesting that selective activation of the specific pathways at an early stage is an essential step for cells towards malignant transformation. Nevertheless, how these genotypic alterations and activation of signaling pathways are interlinked and coordinated to induce malignancy require further experimental validation.

Taken together, this study not only proposed a conserved evolutionary trajectory during preneoplasia initiated by *TP53* LOH, but also provided a paradigm that may allow dissection of genome evolution triggered by other molecular subtype of GC-associated somatic mutations, 2 such as *ARID1A*, *RNF43*^19^, *HNF4A*^20^, and *RHOA*^3,4^.

## Beyond TP53: cell competition and epithelial defense against cancer

Even though the Lowe et al. and Curtis et al. studies concluded that intact *TP53* acts as a barrier, whereas *TP53* LOH initiates a conserved sequential genome evolution. The specified genetic and chromosomal rearrangements differ considerably between the KPC^LOH^ model and *TP53^−/−^* HGOs, thus providing support for the notion that tissue-specific genetic alterations drive tumorigenesis upon TP53 loss. The KPC^LOH^ model allows the intermediate landscapes during benign-to-malignant transforming states *in vivo* to be deciphered, but it is difficult to distinguish the coordinated oncogenic functions of *Kras*, especially for the late-stage malignant phenotypes. Thus, whether the genome reprograming in sole *TP53* LOH cells resembles the evolutionary trajectory observed in the KPC^LOH^ model or induces a distinct genotype-to-phenotype evolution path remains to be clarified. Making this issue even more complex, the preneoplasia cells not only boost their self-proliferative ability, but also deploy various non-autonomous strategies to defeat neighboring cells to eventually evolve as super-competitors and winner clones. Indeed, several independent groups have shown that preneoplastic cells with an *APC* mutation, a hotspot mutation in colon cancer, secrete a protein factor, NOTUM, to act as a WNT pathway antagonist, causing decreased fitness of nearby normal epithelial cells^21–23^.

The current evolutionary trajectories revealed in sole *TP53^−/−^* HGOs are relative “pure paths” without considering the impact from endogenous cell-cell competition. In contrast, it has been increasingly realized that normal epithelial cells are intrinsically able to sense and eliminate neighboring (pre) neoplastic cells, a process recently termed the epithelial defense against cancer (EDAC)^24^ or epithelial surveillance^25,26^. The EDAC has been experimentally illustrated as a non-cell autonomous mechanism to eradicate abnormal cells during the early cancer-initiating stage^16^. A recent study revealed that > 90% esophageal neoplasms were removed *via* EDAC during early clonal evolution before involvement of immune cells^27^. Mechanistically, the epithelial cells also utilize a genomic mutation strategy, just like tumor-initiating cells, to enhance fitness and the ability to outcompete (pre) neoplastic cells. For example, mutation of the Notch pathway in normal esophageal epithelial cells has been shown to drive clonal expansion and therefore restrain early tumorigenesis^28^. Considering that sole TP53 loss can initiate genome arrangements but is not enough for malignant transformation, it is intriguing to imagine that *TP53* LOH may also be utilized as a general mechanism by epithelial cells for evolution to outcompete (pre) neoplastic cells. Because simple manipulation of YAP expression in tumoral and adjacent normal tissues determines the final tumor growth status^29^, the *TP53* LOH-triggered genome evolution may act as a similar “double-edged sword” for genome evolution and cell competition. Furthermore, because YAP and Myc signaling are well-characterized deterministic factors for cell competition and the EDAC^16^, both of which are downstream events of *TP53* LOH, how these events are interlinked to influence cell competition and the EDAC to establish early tumoral clones and shape their evolution path warrants further investigation.

## Conclusions and perspectives

Overall, the identification of ordered genetic evolutionary trajectories upon TP53 deficiency represents a leap forward to understanding how the tumor is initiated and progressively evolves to invasive malignancy. It appears that some deterministic factors, like TP53 loss, shape the evolution pathway for tumor-initiating clones. In the future, additional effort is required to develop more sophisticated *in vivo*, *in vitro*, and *ex vivo* model and tool systems that incorporate cell competition and the EDAC with spatial labelling of involved cells. For example, co-culturing wild-type and *TP53*^−^*^/^*^−^ HGOs and/or transplantation of these modified HGOs back into a mouse model may facilitate more comprehensive and in-depth analysis of genomic and clonal evolution using advanced cell-cell interaction tools^30^. In addition to intercellular competition, the exogenous environmental factors, such as high-fat^31^, low-protein diets^32^, and bacterial pathogens^33^, have also been shown to impair the EDAC ability, resulting in susceptibility for cancer occurrence. Because obesity^34^, alcohol intake^35^, and *Helicobacter pylori* infection^19^ are high-risk factors for GC, how these environmental stimuli influence tumor evolution in the context of TP53 loss represents another interesting open question.
